# Immune Cell Profiling of Peripheral Blood as Signature for Response During Checkpoint Inhibition Across Cancer Types

**DOI:** 10.3389/fonc.2021.558248

**Published:** 2021-03-25

**Authors:** Vinicius Araujo B. de Lima, Morten Hansen, Iben Spanggaard, Kristoffer Rohrberg, Sine Reker Hadrup, Ulrik Lassen, Inge Marie Svane

**Affiliations:** ^1^Department of Oncology, Phase 1 Unit, Rigshospitalet, Copenhagen, Denmark; ^2^National Center for Cancer Immune Therapy, Department of Oncology, Copenhagen University Hospital Herlev, Herlev, Denmark; ^3^Department of Health Technology, Technical University of Denmark, Lyngby, Denmark

**Keywords:** immune checkpoint inhibition, PBMCs, immune signature, prediction, clinical outcome

## Abstract

Despite encouraging results with immune checkpoint inhibition (ICI), a large fraction of cancer patients still does not achieve clinical benefit. Finding predictive markers in the complexity of the tumor microenvironment is a challenging task and often requires invasive procedures. In our study, we looked for putative variables related to treatment benefit among immune cells in peripheral blood across different tumor types treated with ICIs. For that, we included 33 patients with different solid tumors referred to our clinical unit for ICI. Peripheral blood mononuclear cells were isolated at baseline, 6 and 20 weeks after treatment start. Characterization of immune cells was carried out by multi-color flow cytometry. Response to treatment was assessed radiologically by RECIST 1.1. Clinical outcome correlated with a shift towards an effector-like T cell phenotype, PD-1 expression by CD8+T cells, low levels of myeloid-derived suppressor cells and classical monocytes. Dendritic cells seemed also to play a role in terms of survival. From these findings, we hypothesized that patients responding to ICI had already at baseline an immune profile, here called ‘favorable immune periphery’, providing a higher chance of benefitting from ICI. We elaborated an index comprising cell types mentioned above. This signature correlated positively with the likelihood of benefiting from the treatment and ultimately with longer survival. Our study illustrates that patients responding to ICI seem to have a pre-existing immune profile in peripheral blood that favors good outcome. Exploring this signature can help to identify patients likely to achieve benefit from ICI.

## Introduction

The advent of immune checkpoint inhibitors (ICI) as an effective therapeutic modality in oncology has come under spotlight during the last decades, mainly after the first clinical trial showing positive impact of ipilimumab for treatment of patients with metastatic melanoma (1).

Despite encouraging results (2–4), a considerable fraction of patients undergoing ICI still do not benefit from the treatment (5) and primary or acquired ICI resistance is a significant problem that patients and clinicians face on a daily basis. In this context, the field of biomarkers has gained a lot of attention as tool to guide treatment indications and to promote understanding of the biological events taking place in the tumor.

Efforts trying to predict patients likely to respond to ICI are numerous and comprise a myriad of variables spanning from isolated genetic features (e.g. tumor mutational burden, neoepitope load, transcriptomic signatures) (6–9) to multiparametric approaches looking into the tumor microenvironment (TME)’s cellular composition (e.g. immunoscore (10)). Common ground for these methods is the fact that they are tumor tissue-centered, which is reasonable since tumor site harbors the ‘battlefield’ where interactions between cancer and immune system unfold. However, these approaches are limited by the fact that they offer a glimpse of a very dynamic cell milieu and do not take into consideration the effect of non-cellular (11, 12) and extrinsic features (13) that equally can affect T cell function. Another disadvantage is that invasive procedures are required to keep track of changes happening during ICI, which is not always feasible.

Analyses of peripheral blood offer, on the other hand, a non-invasive and simpler alternative to monitor not only biochemical changes (e.g. lactate dehydrogenase, cytokines) but also variations along the treatment in frequencies of immune cells (peripheral blood mononuclear cells - PBMCs) that ultimately can infiltrate the TME and therefore add valuable information in the context of ICI. In this regard, there is evidence supporting that counts of lymphocytes and myeloid subsets in the peripheral blood (14–16), and phenotypical features of circulating T cells [e.g. regulatory T cells (17), CD8+effector-like (18)] seem to correlate with clinical outcome. These analyses, however, have focused on the impact of isolated cell populations on reactivity against neoantigens and/or clinical outcome upon treatment with ICI. Efforts to obtain a more comprehensive knowledge of how different cell types interact with each other during ICI and ultimately affect response patterns is still an ongoing task.

Since most studies addressing the use of PBMCs as predictive tools are restricted to specific tumor types (mainly melanoma) or assess few cell subpopulations at a time, the use of PBMCs requires further investigation prior to implementation in a clinical setting (19–22). Furthermore, predictive power can potentially be increased by involving multiple variables, bearing in mind the multitude of factors preceding and perpetuating immune responses.

In this study, we took a different approach and investigated the immune cell repertoire from peripheral blood (including the influence of both lymphoid and myeloid subsets) of patients with different metastatic solid tumors, in order to gain insight into the impact of differences in distribution of T cells and myeloid cells prior to treatment start and whether changes in these subsets during ICI can shed light on the clinical outcome. We present data indicating that despite possible differences in the PBMC’s composition across patients with different tumor types, there is an underlying preexisting immune signature (here called ‘favorable immune periphery’) among patients likely to benefit from ICI.

As novelty, our study takes into consideration the simultaneous effect of lymphoid and myeloid cells showing that information regarding how peripheral blood is populated may provide patients undergoing ICI with a better starting point.

## Materials and Methods

### Study Design

In order to investigate common immunological features across different tumor types, we designed a basket study.

The protocol was approved by The Danish National Committee on Health Research Ethics (H-16046968) and the Danish Data Protection Agency (RH-2018-44). Informed consent was obtained from all individual participants included in the study prior to collection of biological material. This study was conducted in accordance to 1964 Declaration of Helsinki and its later amendments.

### Cohort

Patients with histologically confirmed metastatic solid tumors referred to our department for ICI treatment were considered as potential candidates for inclusion. Other inclusion criteria were ECOG performance status 0 to 1, age above 18 years, at least one measurable lesion according to response evaluation criteria in solid tumors version 1.1 (RECIST 1.1).

### Response Assessment

Response to the treatment was assessed both clinically and radiologically. Computed Tomography (CT) scan was performed during treatment and assessed with RECIST 1.1. Patients were pooled in two groups referred as ‘benefit’ and ‘no benefit’. The first group comprised individuals who achieved, as best response, either complete response (CR), partial response (PR) or stable disease (SD) with progression free survival (PFS) larger than the median value for the whole cohort (i.e. above than 3 months as described in the result section).

### Blood Samples PBMCs

Immune monitoring was carried out by collection of heparinized blood up to three times over treatment period, i.e. baseline, 6 weeks after treatment start (called ‘T1’) and approximately 20 weeks after treatment initiation(‘T2’). Blood samples were transported in room temperature from ambulatory to our laboratory (elapsed time approximately 2 hours), where they were diluted with phosphate-buffered saline (PBS) (Lonza, Basel, Switzerland), facilitating PBMCs’ by means of centrifugation. Cell count was carried out automatically by Sysmex-XP 300 (Kobe, Japan). Aliquots containing between 2.5x10^6^ to 15x106 cells were cryopreserved in 90% Human AB serum and 10% DMSO. To control the freezing process during the first 24 hours at -80˚C, alcohol-free freezing containers (Cool cell, Biocision) were used. Samples were stored at -140˚C until further use. Overview of biological material is displayed on **Supplementary Table 1**.

In this paper, we focused our flowcytometry analyses on the characterization of subsets of T cells (i.e. CD4+ and CD8+), since they are known to be the final target of checkpoint inhibition. We investigated also the impact of myeloid cell populations known to affect antigen presentation (dendritic cells) and modulate T cell response such as monocytic populations such as classic/non-classic and myeloid derived suppressor cells (MDSCs).

### Phenotyping of PBMCs by Flowcytometry

Cryopreserved PBMCs were thawed in wash buffer (0.5% BSA, 2mM EDTA in PBS) at 37˚C and Fc-receptors blocked by incubation with human IgG (20 µg/mL).

PBMCs were stained in three panels with pre-titrated amounts of premixed reagents: CD3 FITC, CD56 PE, CD11c PE, CD8 PerCP, HLA-DR PerCP, CD27 BV421, CD25 BV421, CD4 BV510, CD28 PE-Cy7, CD3 PE-Cy7, CD19 PE-Cy7, CD127 PE-Cy7, CD45RA APC, CD56 BV510 (all from BD Bioscience, New Jersey, United States), CCR7 PE, PD-1 APC, CD14 BV421 (all from Biolegend, California, United States), CD16 FITC (Dako, Glostrup, Denmark) and NiR live-dead reagent for APC-Cy7 channel (Invitrogen- Thermo Fischer, United States).

Samples were incubated 20 minutes in the dark at 4˚C and then washed prior to acquiring on a FACS Canto II flow cytometer (BD). Data was analyzed on FACSDiva Software version 8.0.1 (BD).

Characterization of CD3+ T cells in both CD4 and CD8 compartments was done by looking at the live singlet events in the PBMC (lymphocyte and monocyte) gate in the forward and side scatter plot. Naïve T cells were further characterized as CCR7+ CD45RA+, central memory (CM) as CCR7+CD45RA-, effector memory as CCR7-CD45RA-, and effector memory RA+ (EMRA) as CCR7-CD45RA+. Exhaustion marker programmed cell death-1 (PD-1) was gated on live CD3+CD8+ cells. Gating strategy for lymphoid cells are displayed on **Supplementary Figure 1**. Myeloid populations were defined as fraction of live singlet cells in the PBMC gate (forward and side scatter plot). Representative gating strategy is displayed on **Supplementary Figure 2**.

### Statistical Analysis

Due to non-normality in flow cytometric data, Mann-Whitney test was used for assessing differences in distributions of cell subpopulations among group of patients according to response pattern. For paired sample analysis, stepwise Wilcoxon test was chosen in order to find whether significant changes occurred and at which timepoint they took place (i.e. from baseline to T1, from T1 to T2 and from baseline to T2). Survival analysis and Cox regression models were done using median value of cell population of interest as threshold for stratification of patients.

All analyses are done using IBM SPSS version 25. Tests were two-sided and p values ≤ 0.05 were considered as significant.

## Results

### Cohort Characteristics

From February 2017 to September 2018, 37 patients with metastatic solid tumors referred to treatment with ICI, either as standard treatment or in a protocol, were screened for inclusion in this study. Four patients were excluded due to withdrawal of informed consent or worsening of performance status (PS) prior to treatment initiation. All patients received ICIs targeting either PD-1 or PD-L1, some in combination with novel therapies targeting potential synergistic targets (**Table 1**). Thirteen different diagnosis were included. The median age at inclusion was 60 years (range 36 to 75) with all patients having PS 0 or 1 by the time of treatment initiation. Prior to inclusion, patients had on average undergone three previous treatment lines. Two patients (no. PB72 and PB74) had previously failed to other ICIs with an interval between failure and inclusion in this study of 12 and 9 months, respectively.

**Table 1:** Cohort characteristics.

Patient no.	General features					Treatment characteristics		Response pattern			
	Gender	Diagnosis	No. of previous treatment lines	PS	Age	Checkpoint inhibition target	No. of immunotherapy cycles	Best achieved RECIST response	OS(months)	PFS(months)	Clinical Benefit
PB2	M	CRC*^a^*	2	1	71	PD-1	4	PD	3.3	1.8	No benefit
PB6	F	Pancreas adenocarcinoma	3	0	75	PD-L1	1	PD	3.1	1.4	No benefit
PB10	M	HCC*^b^*	2	0	43	PD-1+LAG-3	2	SD	4.7	4.0	Benefit
PB12	M	CRC*^a^*	5	1	72	PD-1	7	PD	16.9	6.3	No benefit
PB16	F	Upper GI adenocarcinoma*^c^*	2	1	68	PD-1+LAG-3	4	PD	12.5	1.9	No benefit
PB18	M	Upper GI adenocarcinoma*^c^*	3	1	53	PD-1+LAG-3	1	PD	1.4	0.9	No benefit
PB20	M	HCC*^b^*	2	0	75	PD-1+LAG-3	13	CR	10.8	10.8	Benefit
PB22	M	Urothelial Carcinoma	1	0	62	PD-1	20	PR	15.5	15.5	Benefit
PB24	M	Urothelial Carcinoma	1	0	56	PD-1	22	PR	14.8	14.8	Benefit
PB26	M	CRC*^a^*	3	0	73	PD-L1	6	PD	7.5	4.2	No benefit
PB28	M	NEC*^d^*+NSCLC*^e^*	0	1	68	PD-1	1	PD	6.1	0.7	No benefit
PB30	F	Ovarian cancer	6	0	64	PD-L1	4	SD	7.1	2.1	No benefit
PB32	F	IDC*^f^*	3	0	40	PD-1	11	PR	12.5	12.2	Benefit
PB34	F	IDC*^f^*	1	0	42	PD-1	6	PR	8.6	2.7	Benefit
PB36	F	CRC*^a^*	2	1	57	PD-L1	2	PD	6.3	3.1	No benefit
PB38	F	Ovarian cancer	3	1	49	PD-L1	8	SD	8.7	5.6	Benefit
PB40	F	Head and Neck	0	1	64	PD-1	4	PD	6.3	4.5	No benefit
PB42	M	CRC*^a^*	4	1	66	PD-L1	4	PD	4.0	2.9	No benefit
PB44	F	HCC*^b^*	4	0	58	PD-1+LAG-3	16	CR	24.9	24.9	Benefit
PB46	F	Urothelial Carcinoma	2	1	43	PD-1	1	PD	2.4	0.7	No benefit
PB48	M	Upper GI adenocarcinoma*^c^*	2	0	70	PD-L1	4	PD	6.8	0.6	No benefit
PB50	M	UPT*^g^*	1	1	59	PD-L1	12	SD	9.6	9.6	Benefit
PB54	F	IDC*^f^*	2	0	36	PD-L1	3	PD	4.7	1.5	No benefit
PB56	F	UPT*^g^*	2	1	50	PD-1	1	PD	0.7	0.4	No benefit
PB58	F	Ovarian cancer	3	0	52	PD-L1	3	SD	5.5	4.5	Benefit
PB60	F	MPM*^h^*	3	0	58	PD-1	1	PD	3.3	2.1	No benefit
PB62	F	NSCLC*^e^*	2	1	72	PD-1	4	PD	0.5	2.7	No benefit
PB64	F	Ovarian cancer	7	0	65	PD-L1	1	PD	14.5	1.2	No benefit
PB66	M	NSCLC*^e^*	0	0	60	PD-1	3	PD	4.8	1.8	No benefit
PB68	M	MPM*^h^*	3	0	56	PD-L1	7	SD	4.0	4.0	Benefit
PB70	F	NSCLC*^e^*	2	1	62	PD-1	2	PD	3.3	1.9	No benefit
PB72	F	NSCLC*^e^*	4	0	53	PD-1+CTLA-4	6	PD	2.6	3.7	No benefit
PB74	F	NSCLC*^e^*	5	1	68	PD-1+CTLA-4	4	PD	4.2	1.9	No benefit

^a^Colorectal adenocarcinoma.

^b^Hepatocellular carcinoma.

^c^Upper gastrointestinal adenocarcinoma.

^d^Neuroendocrine carcinoma.

^e^Non-small cell lung cancer-adenocarcinoma.

^f^Invasive ductal carcinoma.

^g^Unknown primary tumor.

^h^Malignant peritoneal mesothelioma.

### Response to the Treatment

Median follow-up period was 7.4 months. The overall median PFS was 81 days (95%CI 46-116) and the median OS was 188 days (95%CI 148-227). We did not observe any statistically significant correlation between the number of treatments received prior to initiation of ICI and clinical outcome (Mann-Whitney test, p= 0.15; Fisher exact test assuming cut-off of three treatments with p=0.69). Neither did we observe any difference in benefit between the group receiving combination therapy and the group receiving monotherapy (Fischer exact test p=0.29). Best tumor size variation during therapy for each patient can be seen on waterfall plot - **Supplementary Figure 3**.

### Exploring the T Cell Phenotype in Peripheral Blood

In order to get insight into how treatment impacted the functional repertoire of T cells, we performed phenotyping of cryopreserved PBMCs collected at 3 time points, i.e. baseline, T1 and T2. Differences in distribution of several subsets of T cells were investigated at each time point. Stepwise changes from baseline to T1, from T1 to T2 and baseline to T2 were based on paired samples available for 26 patients (9 presenting treatment benefit), 12 patients (7 with benefit) and 11 patients (6 with benefit), respectively.

In the current study, we hypothesized that patients likely to respond to ICI have a peripheral lymphoid and myeloid cell signature characterized by high cytotoxic activity, presence of treatment target on T cells (i.e. expression of PD-1), high antigen presentation, and low counts of immunosuppressive cells.

To address the first premise of our hypothesis, we investigated the functional subsets of circulating CD4^+^ and CD8^+^ T cells by looking into expression of CD45RA, CCR7, CD25 and expression of PD-1.

In the CD4 compartment, T cells were mainly represented by naïve and central memory (CM) phenotypes at all time points, regardless of response pattern. We also observed that percentages of different CD4^+^ subsets at each time point were similar between patients that benefited from the treatment and those that did not (p>0.05 - **Supplementary Figure 4A**). Paired samples analysis revealed among the patients benefiting from ICI a statistically significant decrease of CD4^+^ effector memory (EM) from baseline to T1(median 21.7% vs 19.6%, p=0.008) whereas CD4^+^ effector memory RA^+^ (EMRA) increase from baseline to T1, and overall from baseline to T2 (Wilcoxon test p=0.024 and p=0.043, respectively-**Supplementary Figure 4B**).

Unlike CD4 compartment, CD8^+^ T cells had a predominant effector/cytotoxic phenotype (i.e. EM and EMRA) at all time points (**Supplementary Figure 5A**). Paired analysis comprising baseline and T1 samples revealed a slight but non-significant increase of the effector subset (i.e. sum of EM and EMRA) from 70.4% to 72.6% during treatment (p=0.052). Variations in levels of EMRA CD8+ T cells in the benefit and no benefit group were not statistically significant - **Supplementary Figure 5B**.

**Figure 1:**
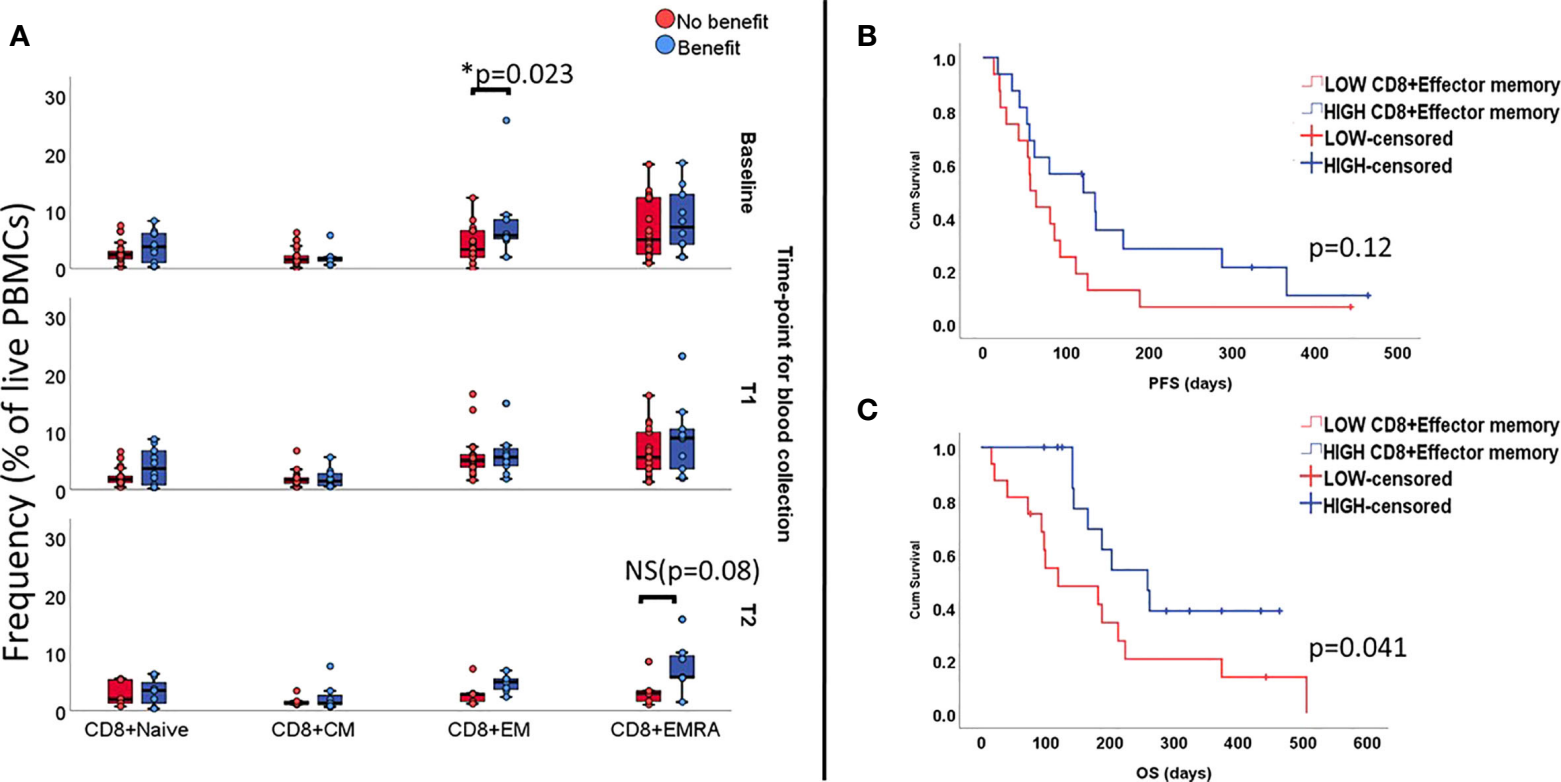
Levels of functional subsets of CD8+T cells as fraction of live PBMCs at all three time-points (unpaired samples). Baseline analysis are carried out on 32 samples (10 deriving from patients benefiting from treatment), T1 comprises 27 samples (10 presenting benefit) and T2 comprises 12 samples (7 with treatment benefit). Section **(A)** shows an association with benefit during ICI treatment and levels of CD8+EM T cells at baseline and increased levels of EMRA in the group benefiting from treatment at late follow-up, with marginal p value. Effect of CD8+EM T cells on survival is displayed on **(B)** for PFS and **(C)** for OS.

Next, we proceeded to investigate to which extent lymphocyte subsets as a fraction of the PBMC gate are associated with clinical outcome. Patients with clinical benefit presented higher percentage of CD8^+^ EM T cells at baseline (5.7% vs 3.3%, p=0.023). In the same group of patients, we found that, over time, the phenotype became more effector-like with increasing numbers of circulating CD8^+^EMRA at T1 which, despite a decrease at T2, still seemed to be higher when compared to patients not benefiting from ICI (5.8% vs. 2.9%; p=0.08, **Figure 1A**).

In terms of survival, patients with high numbers of circulating CD8^+^EM T cells at baseline(i.e. above median value), did not have longer median PFS (121days vs. 57 days, Log rank p=0.12 **Figure 1B**), but the observed median OS was longer (259 days vs.120 days, Log Rank p= 0.04, **Figure 1C**).

**Figure 2:**
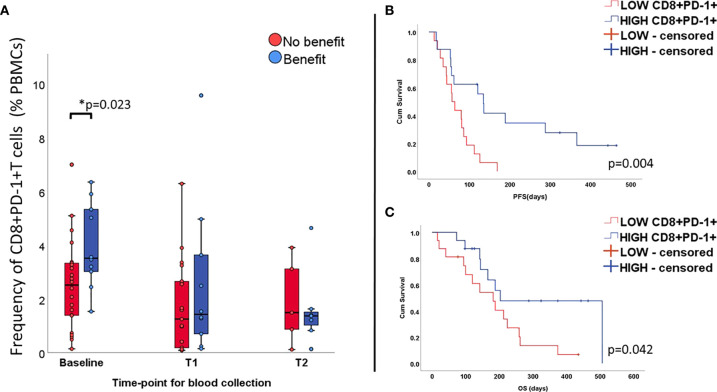
Percentage of CD8+T cells expressing PD-1 at all time-points (unpaired samples) and correlation with outcome. Levels of CD8+PD-1+ T cells are higher at baseline in the group benefiting from the treatment **(A)** and correlates positively with longer PFS **(B)** and OS **(C)**.

Since all patients in our cohort received treatment blocking the PD-1/PD-L1 axis, we then proceeded to investigate whether the expression of PD-1 by circulating CD8^+^T cells correlated with outcome. Results for baseline samples demonstrated that patients with relatively high values of CD8^+^PD-1^+^T cells in the peripheral blood (i.e. above median value) benefited most from the treatment (p=0.023, **Figure 2A**) presenting extended PFS (135 days vs. 57 days; LogRank p=0.004, **Figure 2B**) and OS (203 days vs 188 days; Log Rank p=0.042, **Figure 2C**). In univariate Cox regression, baseline high counts of CD8^+^PD-1^+^T cells correlated significantly to longer PFS (HR=0.31, p=0.006; 95%CI 0.12-0.71) but no solid trend regarding longer OS was verified (HR 0.4, p=0.051; 95%CI 0.16-1.0). No difference in CD8^+^PD-1^+^ T cell levels was observed for follow-up samples.

It has also been reported that expression of PD-1 and effects deriving from blockade of this axis are tightly related to expression of CD28 by CD8^+^ T cells. In our data, we observed that the number of CD8^+^ T cells expressing this molecule increased significantly upon treatment initiation, regardless of clinical outcome (**Supplementary Figure 6**).

Further, we investigated the impact of regulatory T cells (CD3^+^CD4^+^CD25^+^CD127low). Even though, this cell subset is known for damping immune responses, levels of Tregs have not served as a unifying marker for response to therapy. In our study, we found that, at all timepoints, T regs were elevated among patients benefiting from treatment, reaching statistical significance level at T2 (p=0.045; **Supplementary Figure 7**). Longitudinal measurements showed in benefiting patients a gradual and statistically significant increase in the levels of the T reg population, especially from baseline to T2 (median 7.7% to 11.6%, p= 0.046; **Supplementary Figure 7**).

### Role of Myeloid Cells

**Figure 3:**
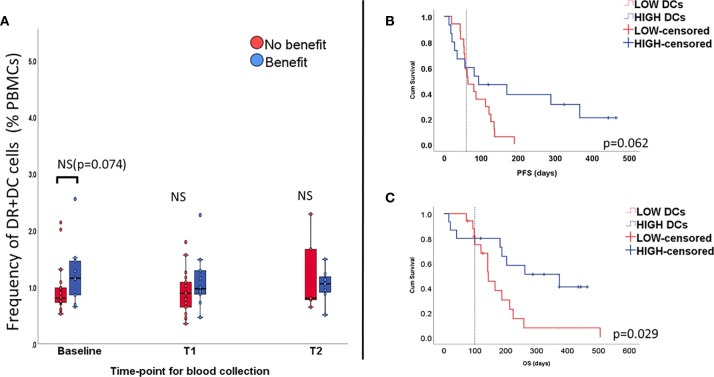
Percentage of dendritic cells (DCs) all time-points (unpaired samples) and correlation with outcome. Levels of DCs seem to be elevated at baseline in the group benefiting from the treatment, but not at longitudinal measurements **(A)**. High levels of DCs seem to affect PFS **(B)** and OS **(C)**.

The association of myeloid cell levels and clinical outcome and survival was addressed by investigating dendritic cells (DC), monocytic MDSC (mMDSCs) and monocyte subpopulations. DCs were chosen due to the important role they play in terms of antigen presentation. This cell subset was gated as the fraction of PBMCs co-expressing CD11c and HLA-DR, and lacking CD14, CD16 and CD56. We found a trend between treatment benefit and abundance of DCs at baseline, however not statistically significant (1.15% vs. 0.8%, p=0.074, **Figure 3A**). Over time, variations in percentage of this cell type were small and not significant. Yet, survival analysis appeared to show impact of DC levels at baseline on PFS (Log Rank p= 0.062, **Figure 3B**) and OS (p=0.029, **Figure 3C**). High counts of DCs (i.e. above median value) also correlated with longer PFS (HR=0.14, p=0.03 95%CI 0.026-0.83) but not OS (HR=0.18, p=0.087 95%CI 0.026-1.28).

**Figure 4:**
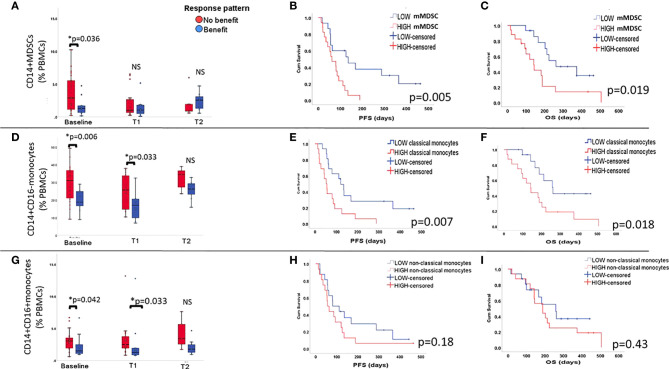
Frequency of CD14^+^ myeloid populations is inversely associated with benefit and survival. Percentages of mMDSCs **(A)**, classical monocytes **(D)** and non-classical monocytes **(G)** out of alive PBMCs (y-axis) are, already at baseline, elevated in the group of patients that did not achieve benefit during ICI treatment. Effects on survival for the respective myeloid subsets are displayed on **(B, E, H)** for PFS and **(C, F, I)** for OS. Overall, classical monocytes and mMDSCs affected negatively survival times. No effect on PFS nor OS was observed for non-classical monocytes.

Other cell types of myeloid lineage have been reported as immune suppressive elements in TME. To investigate whether this applies for the periphery we explored the impact of mMDSC and monocyte populations. In this regard, we found that high baseline levels (i.e. superior to the median value) of mMDSCs (CD14^+^ CD3^-^ CD19^-^ HLA-DR^low^, CD56^-^), classical (CD14^+^CD16^-^HLA-DR^+^), and non-classical (CD14^+^CD16^+^ HLA-DR^+^) monocytes were associated with poorer outcome (p= 0.036, 0.006, 0.042, respectively - **Figures 4A, D** and **G**). Changes of these populations during therapy did not reach statistically significant levels (Wilcoxon test p>0.05).

Longer PFS and OS were associated with low baseline levels of mMDSC (LogRank p=0.005 and 0.019, **Figures 4B, C****)** and classical monocytes (Log Rank p=0.007 and 0.018, **Figures 4E, F****)**. Non-classical monocytes did not seem to have any impact on survival (**Figures 4H, I****)**.

Univariate Cox regression models were then used to investigate the effects of mMDSC and classical monocytes on survival. We found that high baseline counts of circulating mMDSC were associated with shorter PFS (p= 0.008, HR= 3.15, 95%CI 1.35-7.35) and OS (p=0.025, HR=2.78, 95%CI 1.13-6.8). For classical monocytes, we also found a similar trend both in terms of PFS (p=0.01, HR= 2.7, 95%CI 1.2-6.0) and OS (p=0.024, HR= 2.85, 95%CI 1.14-7.1).

### The ‘Favorable Immune Periphery’ Concept

The previous analysis showed that several immune cell types in peripheral blood have the potential to affect how patients undergoing ICI benefit from the treatment in terms of clinical response and survival.

**Figure 5:**
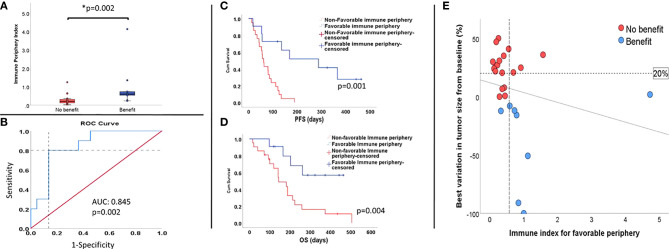
The favorable immune periphery index and its impact on clinical outcome. Patients benefiting from ICI treatment presented a higher index **(A)**. Threshold for favorable immune periphery index was determined by ROC, assuming the highest sensitivity and specificity as possible **(B)** represented by the point where dashed lines cross each other. Patients with a favorable immune periphery had longer PFS **(C)** and OS **(D)**. On **(E)**, the scatter plot depicts correlation between best tumor variation and immune index based on baseline samples. Two parameters are inversely correlated (Spearman’s ρ= -0.40; p=0.043). Vertical dashed line represents the threshold for immune index determined by ROC. Horizontal dashed line represents 20% limit for tumor growth and distinguishes patients who progressed (zone above 20%) from those that did not (area bellow below 20%). Patients that did not benefit from the treatment tend to cluster below the threshold for favorable periphery index.

Overall, we noted that baseline counts of CD8^+^ T cells with effector memory phenotype, CD8^+^T cells expressing PD-1 and DC cells as a fraction of total live PBMCs seemed to correlate positively to response and survival, whereas mMDSCs and classical monocytes as a fraction of total live PBMCs seemed to be associated with poor clinical outcome. Based on these results, we hypothesized that patients benefitting from checkpoint inhibition have already at baseline a favorable immune signature that upon ICI could lead to improved clinical outcome. Bearing this in mind, we elaborated a numerical index where relative counts (i.e. percentage of live PBMCs) of CD8^+^PD1^+^, CD8+effector memory cells and DCs were entered as numerator and mMDSCs and classical monocytes were entered as denominator (Equation 1 - **Supplementary Material**). The geometric mean values for both parts of the fraction where used to minimize the effect of outliers. Patients achieving clinical benefit had a significantly higher index, suggesting a prevalence of factors promoting immune antitumor activity, p=0.002 (**Figure 5A**). Reiterating this, we noted that our index significantly correlated with treatment related change in tumor size (Spearman’s rho: -0.40, p=0.043, **Figure 5E**).

Sensitivity and specificity of the index were tested by looking at receiver operational characteristic curve (ROC). A cut off for the index was determined where sensitivity and specificity were equally as high as possible (dashed lines on **Figure 5B**).

Patients were subsequently pooled in two groups, namely high (i.e. favorable immune periphery; n=11) and low ‘favorable immune periphery’-index (i.e. non-favorable immune periphery, n=21). The high favorable-index patients presented longer PFS (288 days vs 62 days; p= 0.001, **Figure 5C**) and OS (p=0.013, median survival not reached, **Figure 5D**), and equally exhibited reduced risk of progression (HR=0.18, p=0.002, 95%CI 0.065-0.52) and death (HR=0.27, p=0.021, 95%CI 0.09-0.82).

## Discussion

Immunotherapy with checkpoint blockade is a fast-growing field in the landscape of cancer treatment. However, despite documented benefit in terms of survival, the effect is only seen in a smaller fraction of patients which most likely reflects the multiple features that directly and indirectly affect T cell reactivity (23). Studies trying to find predictive biomarkers for response have broadly focused on variables spanning from genetic determinants, cell composition in the tumor microenvironment to markers in the peripheral blood and microbiome (22, 24). Majority of these approaches have focused on specific tumor types and have taken into consideration few factors at a time. With the increasing number of basket trials trying to find best therapeutic options and which patients are likely to benefit from ICI, there is an imperative need of multiparametric biomarker models capable of assessing broader and more heterogenous cohort of patients. Studies conducted so far using circulating immune cells have mainly investigated role played by T cells upon ICI, despite the growing evidence that other cell types might equally influence how patients respond to immunotherapy.

Bearing this in mind, we investigated by means of conventional flow cytometry and across tumor types, the peripheral immune cell repertoire (both lymphoid and myeloid subsets) of patients undergoing ICI, how the treatment affects this immune profile and whether these changes can possibly impact clinical outcome.

Our results suggest that patients benefiting from ICI seem to have a pre-treatment immunological profile (here called ‘favorable immune periphery’) entailing: high levels of T cells carrying treatment target (i.e. PD-1) and with cytotoxic potential; moreover, a periphery where cells involved in antigen processing/presentation (DCs) were present and where immunosuppressive effects (mMDSC, CD14^+^CD16^-^monocytes) were not prominent. Such changes were not observed to be reliant of tumor type. By pooling patients according to the two possible scenarios (favorable versus non-favorable immune periphery), we could distinguish patients with high chance of achieving benefit from those where treatment did not improve outcome.

In the lymphoid lineage, we focused our analysis on T cells, since they are the ultimate target of ICI. For the CD4^+^ compartment we observed that, T cells mainly expressed a naïve or central memory phenotype. Percentages of these functional subsets remained stable over time, while a decrease in EM and an increase in EMRA^+^ CD4^+^ was seen in patients responding to the treatment. CD4^+^T cells play a role not well-explored in checkpoint inhibition as cellular cytotoxic activity usually is attributed to CD8^+^ T cells. However, there is growing evidence supporting the role played by CD4^+^T cells in cancer immunology. Active circulating CD4^+^Th cells (i.e. expressing PD-1 and TIM-3 or CD62L^low^) have, for instance, been shown to correlate with a better outcome in cohorts of breast (25) and lung cancer (26).

Intriguingly, we found an increased level of T regs in patients benefiting from ICI at late follow-up as well as expansion of T regs during treatment, possibly as an attempt to keep homeostasis upon the successful activation of T cells during ICI among patients responding to the treatment Proliferation of CD4^+^CD25^+^T regs following treatment with checkpoint inhibition has been previously observed mainly in patients treated with anti-CTLA-4 (27–29), with some data showing that T regs expanded upon ICI targeting CTLA-4 expressed FoxP3 but did not release IL-2, inferring that they were in fact ‘bona fide’-T regs (30). On the other hand, studies assessing T regs under PD-1/PD-L1 blockade show a distinct downregulation of FoxP3 for melanoma (31) while in gliomas exhausted T regs with high expression of PD-1 failing to suppress effector T cell proliferation accumulated (32). These diverse results demonstrate the heterogeneity of T regs and that further studies are needed to get a clearer picture of their role in immune activation during ICI.

Contrarily to the observations for CD4^+^T cells, a large fraction of CD8^+^ T cells exhibited a more effector-like phenotype and clinical benefit was associated with higher baseline levels of EM and, at late follow-up, with increased levels of CD8^+^EMRA, a functional subset known to produce granzymes, perforin and INF-gamma (33). The correlation between a shift towards a more effector-like phenotype and positive outcome has also been demonstrated by Kamphorst et al (18). in a NSCLC cohort undergoing PD-1 targeted therapy where efficacy was associated with an increase in CD8^+^ effector T cells in peripheral blood within the first 4 weeks. Positive effects of CD8+EM T cells on survival during treatment with ICI have also been shown in patients with metastatic melanoma (34).

Although outcome seemed to be linked to a more cytotoxic CD8 phenotype, we did not note any significant correlation to progression-free survival. A possible explanation is that, even though, phenotypically, these T cells have cytolytic activity, that does not necessarily imply that the circulating CD8^+^T cells in question are indeed only tumor specific. As a matter of fact, Kamphorst et al (18). highlights that the tumor reactive CD8^+^T cells expanding in peripheral blood expressed Ki67 and were positive for PD-1^+^. Also, Gros et al. find that expression of PD-1 could be used to determinate tumor specific reactive CD8^+^T cells in the tumor (35) as well as in peripheral blood in melanoma patients (36). In line with these findings our results suggest that patients benefiting from ICI have high counts of CD8^+^T cells expressing PD-1 at baseline. Thus, expression of PD-1 indicates tumor reactive T cells and by blocking PD-1/PD-L1, specific antitumor activity is unleashed initially at periphery and later in the tumor as CTLs move from blood stream into the TME. As a matter of fact, a recent work by Yost and colleagues has shown that T cells responses arising upon PD-1 blockade are mainly due to new T cell clones entering the tumor rather than the already existing T cells in TME (37).

Follow-up measurements indicated a reduction of the PD-1 positive CD8 population during therapy which could be explained by the fact that PD-1 staining was affected by the therapeutic antibody given during ICI treatment, as previously demonstrated by Zelba et al. (38). Another reason could also be that our second blood collection (approximately 6 weeks after treatment start) does not time with the moment when expansion in relative numbers of this cell population took place or due to migration of these tumor specific T cells to TME.

Reactivity of T cells is also reliant on the crosstalk between lymphoid and non-lymphoid cell types. An important part of cytotoxicity lies on antigen presentation and activation provided by APCs, which in the peripheral blood, are represented by a broader class of myeloid cells. We covered the role of antigen presentation by looking into myeloid DCs as they are capable of antigen uptake/processing/presentation and thereby promote priming of naïve T cells (39). In line with other studies (40, 41), DCs were scarcely represented at the periphery. Circulating myeloid DCs tended already at baseline to be slightly more abundant among patients experiencing treatment benefit, however, not reaching statistical significance. The lack of impact on response, when employing DCs as a single parameter, could likely be attributed to their paucity in peripheral blood

The relevance of the myeloid lineage in the context of ICI is still noteworthy. Several studies have provided strong evidence that these cells, although not directly involved in the immunological synapse, can still influence processes triggered upon antigen recognition (20, 42, 43). Sun et al. has recently demonstrated that abrogation of mMDSCs’ trafficking into the TME using chemokine receptor inhibitors led to improved T cell infiltration and subsequent tumor cell killing (44). In our study, high counts of mMDSCs prior to treatment culminated with shortened PFS and OS. Our analyses corroborate the immunosuppression associated with this myeloid fraction (20, 44, 45) and similar trend for classical monocytes. Several studies have shown that the presence of macrophages in the tumor is, in general, associated with a poor prognosis (46). A recent work by Krieg et al. showed, however, that the circulating counterpart of classical monocytes (CD14^+^CD16^-^HLA-DR+) actually seem to be associated with better response in melanoma patients undergoing anti-PD-1 therapy (16), which is in contrast to our findings. Reasons for these divergent results could be difference in patient cohort composition (several histologies versus one single immunogenic tumor type) and the method used of characterizing cell phenotype (conventional Flow cytometry versus Mass Cytometry - CyTOF).

Overall, we did not find any statistically significant increase of specific myeloid and lymphoid subsets (except for T regs, as previously discussed) from baseline to later time-points. Even though, we could not see clear expansion of certain cell subsets among patients benefitting ICI, we did find that the effect on clinical outcome of the cells populations we looked into was also quite similar to what has been previously described, which is noteworthy considering the heterogeneous cohort composition. Moreover, we found a persistent pattern showing that patients who benefitted from the treatment seem to have at baseline a preexisting immune signature in peripheral blood which makes the basis for our predictive index.

Even though our study has the clear limitation of sample size, the exploratory character, cohort’s heterogenicity and need of validation in bigger cohorts it brings as novelty the fact that compiling the activity of lymphoid and myeloid subsets suggests that, also across tumor types, patients benefiting from ICI seem to have a pre-existing favorable immune signature in peripheral blood that provides a better biological starting point and ultimately better outcome. Furthermore, different cell types taken as explanatory variables cannot stand alone when predicting response to ICI, which underscores the complexity of mechanisms preceding and sustaining T cell activation. Therefore, designing multiparametric peripheral immunity models like ours and validating them in a bigger scale of patients is important to enable more precise predictive tools for clinical decision making.

## Data Availability Statement

The original contributions presented in the study are included in the article/**Supplementary Material**. Further inquiries can be directed to the corresponding authors.

## Authors Contributions

VL: study design, idea development, inclusion of patients, collection of biological material and clinical data, flow cytometry panel design(secondary), statistical analysis and data visualization, manuscript writing. MH: Primarily flow cytometry panel design, gating and export of flow cytometry data. Review of manuscript. IS: facilitating patient recruitment, review of manuscript. KR: facilitating patient recruitment, review of manuscript. SH: study design, review of manuscript. UL: study design, patient recruitment and logistics related to transport of biological material, review of manuscript I-MS: study design, laboratory facilities, review of manuscript. All authors contributed to the article and approved the submitted version.

## Ethics Statement

The protocol was approved by The Danish National Committee on Health Research Ethics (H-16046968) and the Danish Data Protection Agency (RH-2018-44). Informed consent was obtained from all individual participants included in the study prior to collection of biological material. This study was conducted in accordance to 1964 Declaration of Helsinki and its later amendments.

## Funding

This study was only possible due to financial support granted by The Danish Cancer Society (R149-A10123) and Preben & Anna Simonsens Fond (Grant number 021892-0009).

## Conflict of Interest

The authors declare that the research was conducted in the absence of any commercial or financial relationships that could be construed as a potential conflict of interest.
