# Supplementary material for: Immune Cell Profiling of Peripheral Blood as Signature for Response During Checkpoint Inhibition Across Cancer Types
Source: Front Oncol. 2021 Mar 25;11:558248. doi: 10.3389/fonc.2021.558248 (PMC8027233; doi:10.3389/fonc.2021.558248)
Supplement: Supplementary file 1 [file DataSheet_1.docx]

Supplementary – Frontiers in Oncology (submitted in 2020) – Vinicius A. B. de Lima et al.

| Patient no. | Blood sample- baseline | Blood sample T1 | Blood sample T2 | Best RECIST response | Clinical Benefit |
| --- | --- | --- | --- | --- | --- |
| PB2 | yes | yes | no | PD | No benefit |
| PB6 | yes | no | no | PD | No benefit |
| PB10 | yes | no | no | SD | Benefit |
| PB12 | yes | yes | yes | PD | No benefit |
| PB16 | yes | yes | yes | PD | No benefit |
| PB18 | yes | no | no | PD | No benefit |
| PB20 | yes | yes | yes | CR | Benefit |
| PB22 | yes | yes | yes | PR | Benefit |
| PB24 | yes | yes | no | PR | Benefit |
| PB26 | yes | yes | yes | PD | No benefit |
| PB28 | yes | no | no | PD | No benefit |
| PB30 | yes | yes | yes | SD | No benefit |
| PB32 | yes | yes | yes | PR | Benefit |
| PB34 | yes | yes | yes | PR | Benefit |
| PB36 | yes | yes | no | PD | No benefit |
| PB38 | yes | yes | yes | SD | Benefit |
| PB40 | yes | yes | no | PD | No benefit |
| PB42 | yes | yes | no | PD | No benefit |
| PB44 | no | yes | yes | CR | Benefit |
| PB46 | yes | no | no | PD | No benefit |
| PB48 | yes | yes | yes | PD | No benefit |
| PB50 | yes | yes | yes | SD | Benefit |
| PB54 | yes | yes | no | PD | No benefit |
| PB56 | yes | no | no | PD | No benefit |
| PB58 | yes | yes | no | SD | Benefit |
| PB60 | yes | yes | no | PD | No benefit |
| PB62 | yes | yes | no | PD | No benefit |
| PB64 | yes | yes | no | PD | No benefit |
| PB66 | yes | yes | no | PD | No benefit |
| PB68 | yes | yes | no | SD | Benefit |
| PB70 | yes | yes | no | PD | No benefit |
| PB72 | yes | yes | no | PD | No benefit |
| PB74 | yes | yes | no | PD | No benefit |

Supplementary Table1**.** Overview of used biological material per patient and response pattern


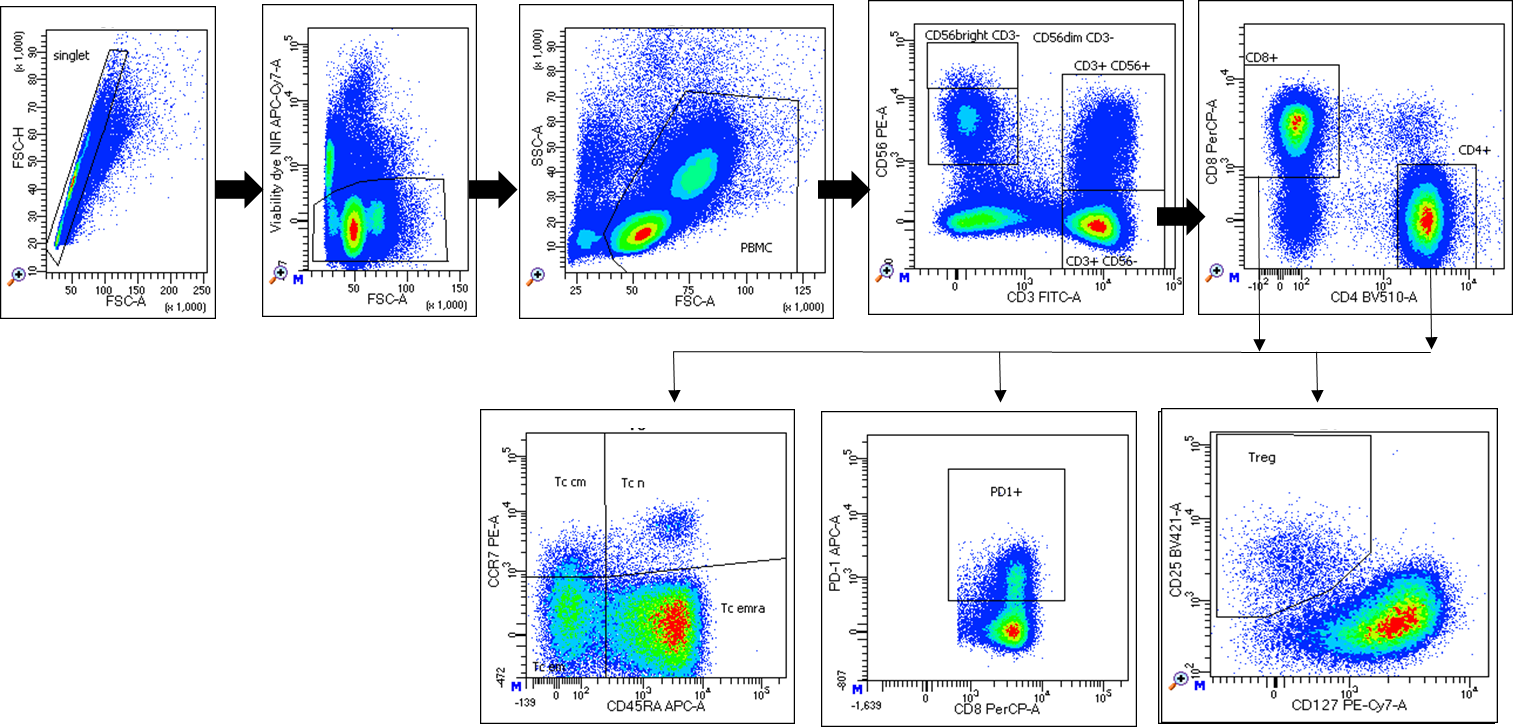


Supplementary Figure 1. Gating strategy on lymphoid subfractions of peripheral blood mononuclear cell analysis. The initial gate was set in the FSC-H/FSC-A plot identifying singlets. From the singlets, dead cells were gated out (NIR APC-Cy7 x FSC-A plot). Subsequently, PBMC gate comprising lymphoid and myeloid subsets was set in the SSC/FSC plot. T cells were identified by expression of CD3. T cells were then divided according to expression of CD4 and CD8. In the CD4 and CD8 gates, T cells were further subclassified according to expression of CCR7 and CD45RA into: Naïve T (CCR7+ CD45RA+), central memory (CCR7+CD45RA-), effector memory (CCR7-CD45RA-), and effector memory RA+ (CCR7-CD45RA+). CD8 compartment was further characterized according to the expression of PD-1. T regs were defined by expression of CD25 and low expression of CD127 among CD4+ T cells.


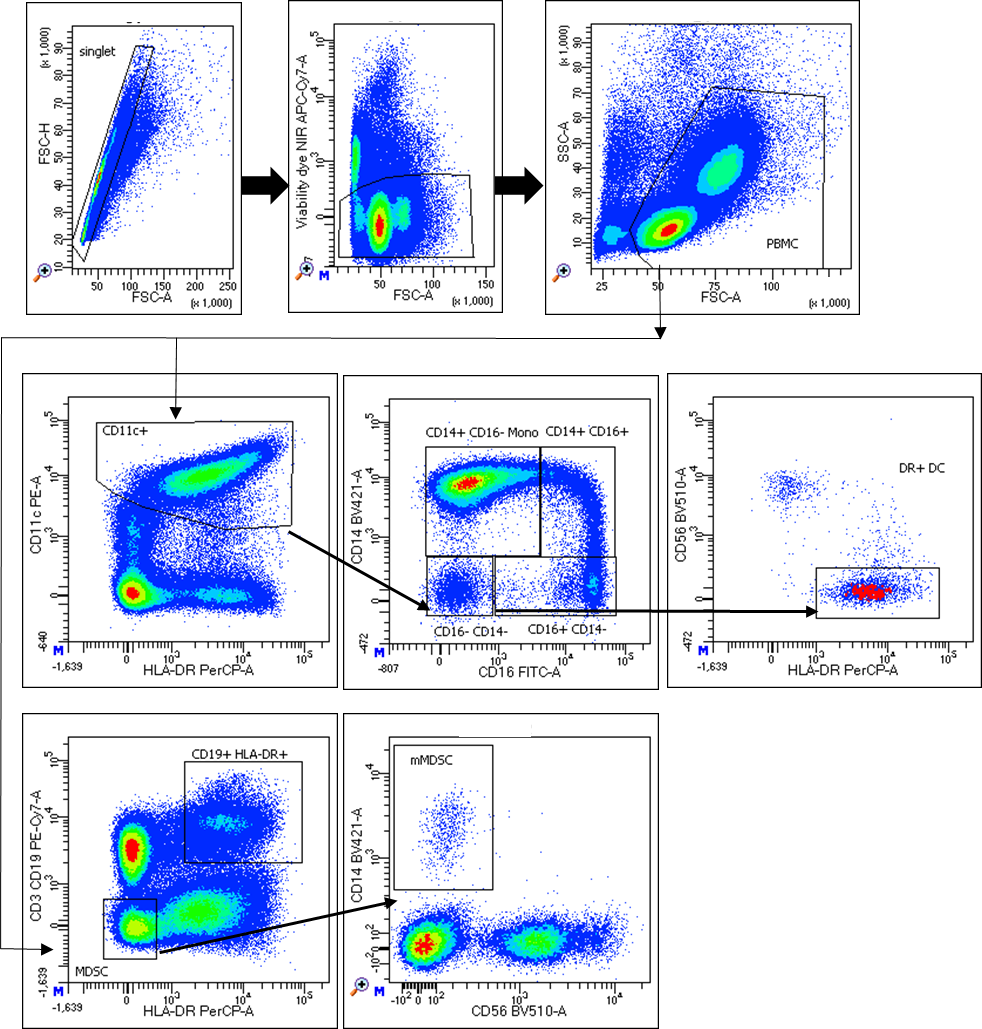


Supplementary Figure 2. Gating strategy on myeloid subfractions of peripheral blood mononuclear cell analysis. The initial gate was set in the FSC-H/FSC-A plot identifying singlets. From the singlets, dead cells were gated out (NIR APC-Cy7 x FSC-A plot). Subsequently, PBMC gate comprising lymphoid and myeloid subsets was set in the SSC/FSC plot. Myeloid cells in the PBMC gate were identified by expression of CD11c and HLA-DR. These cells were then stratified according to the expression of CD14 and CD16. Classical monocytes were defined as CD14^+^CD16^-^ whereas non-classical monocytes were double positive for these markers. Dendritic cells were characterized by lacked expression of CD14, CD16 and positivity for HLA-DR and CD11c. Monocytic MDSCs (mMDSC) were defined as being CD14^+^ and lineage (CD3, CD19), CD56 and HLA-DR negative


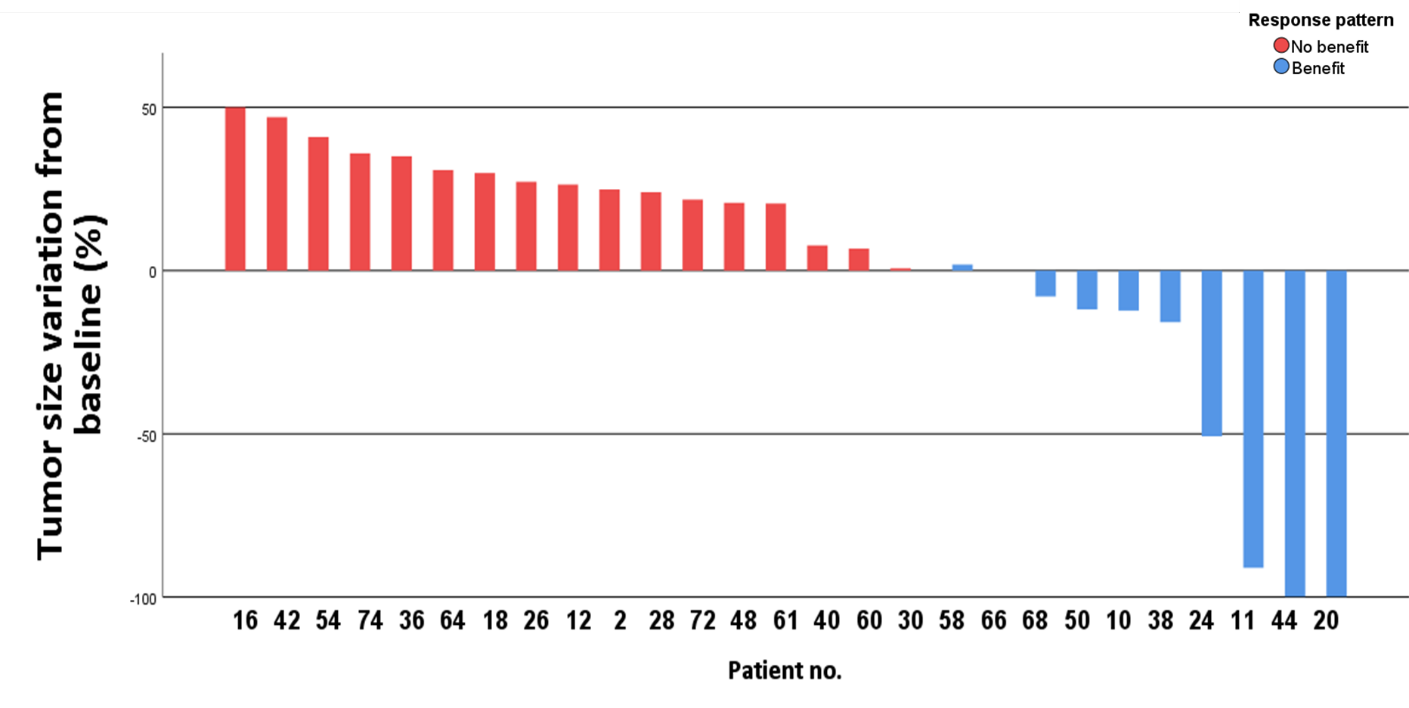


Supplementary Figure 3. Waterfall plot showing best tumor size variation from baseline CT scans.


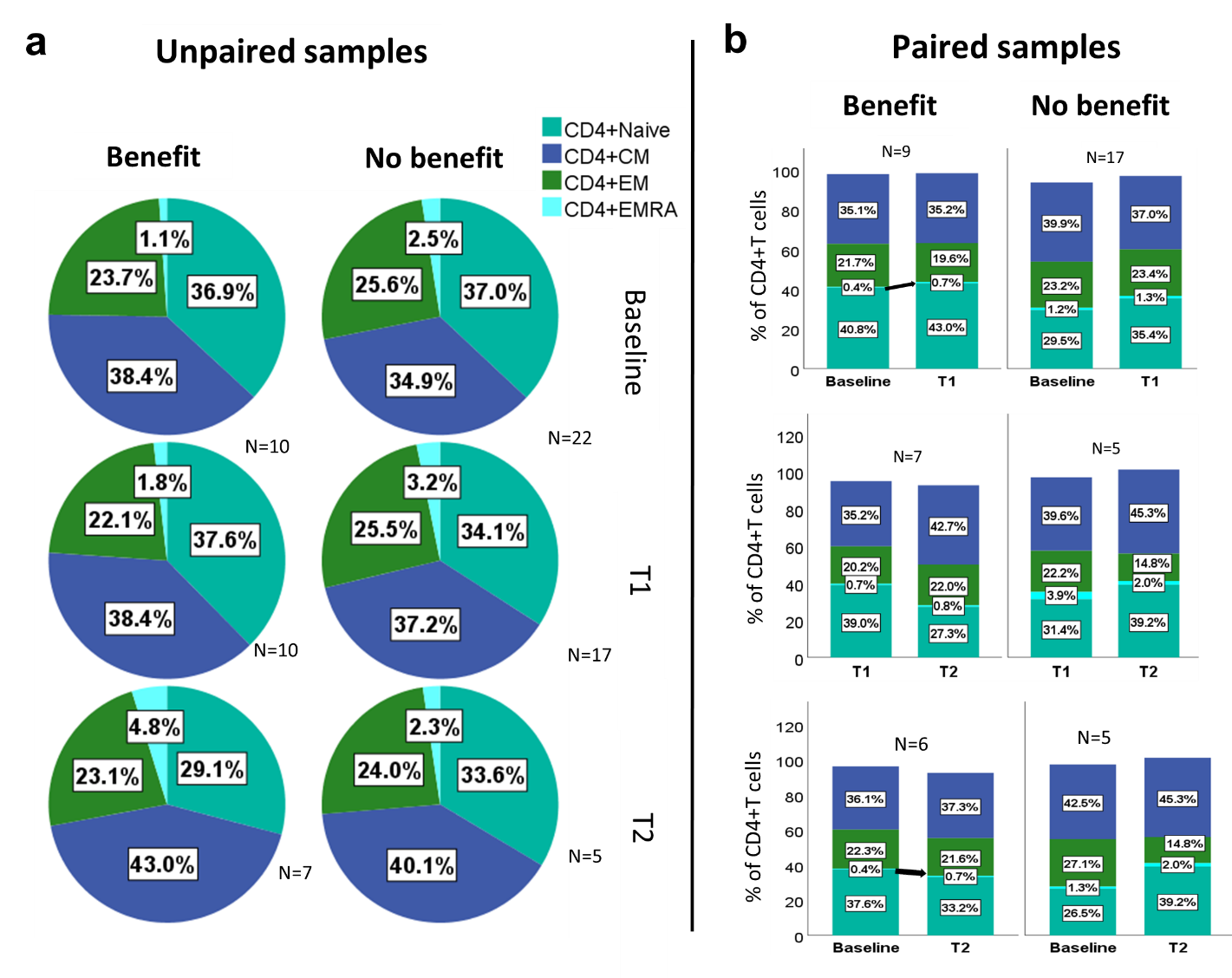


Supplementary Figure 4. Subsets of CD4+ T cells according to CCR7 and CD45RA at all time points. In (a), numbers in the pie charts represents mean values of each subpopulation. Mann-Whitney test for median values was not significant for any CD4+subset. In (b). Numbers represent median values, therefore are not expected to sum up 100%.


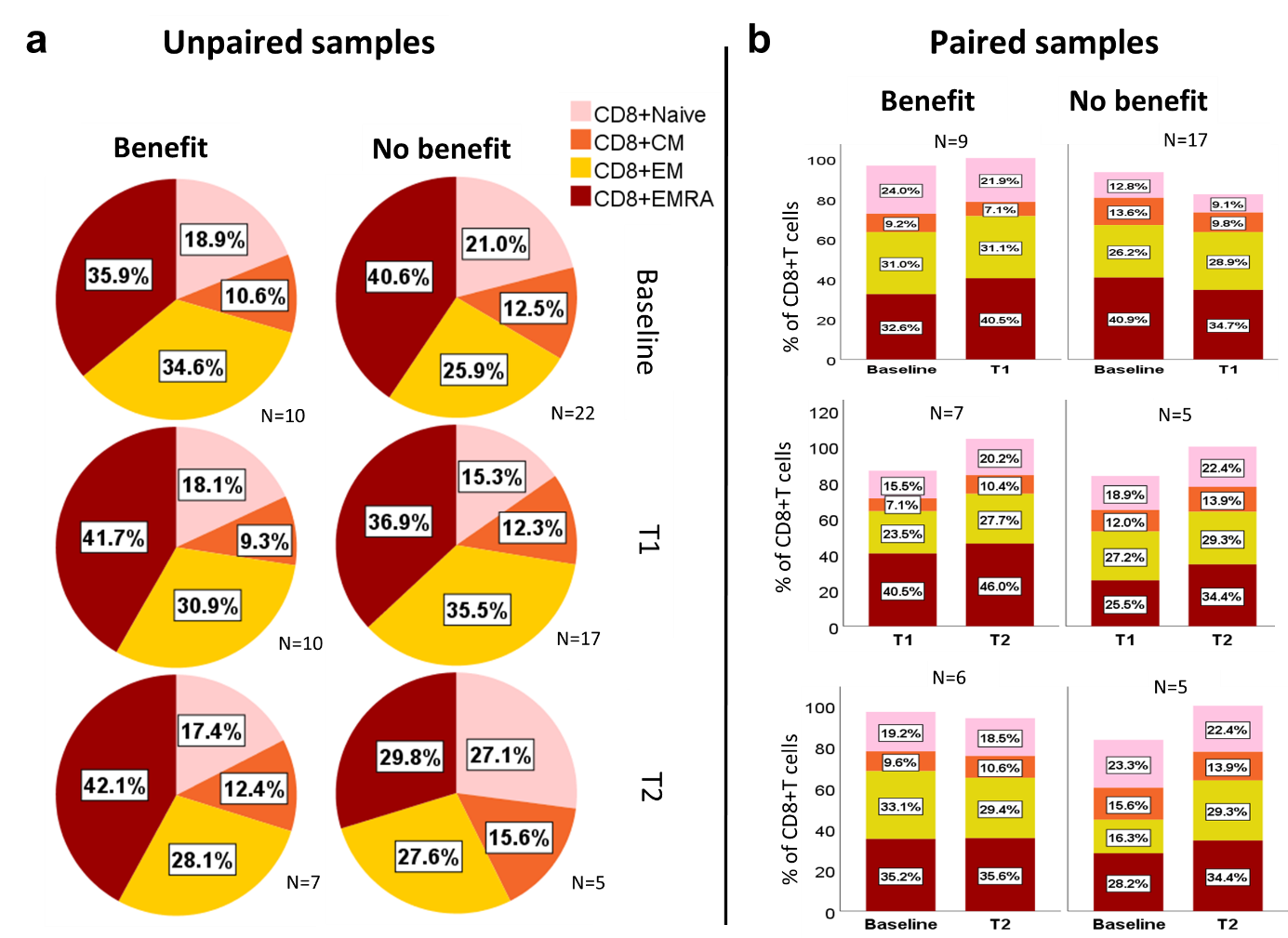


Supplementary Figure 5. Subsets of CD8+ T cells according to CCR7 and CD45RA at all time points. In (a), numbers in the pie charts represents mean values of each subpopulation. Mann-Whitney test for median values was not significant for any CD8+subset. In (b) numbers represent median values, therefore are not expected to sum up 100%.


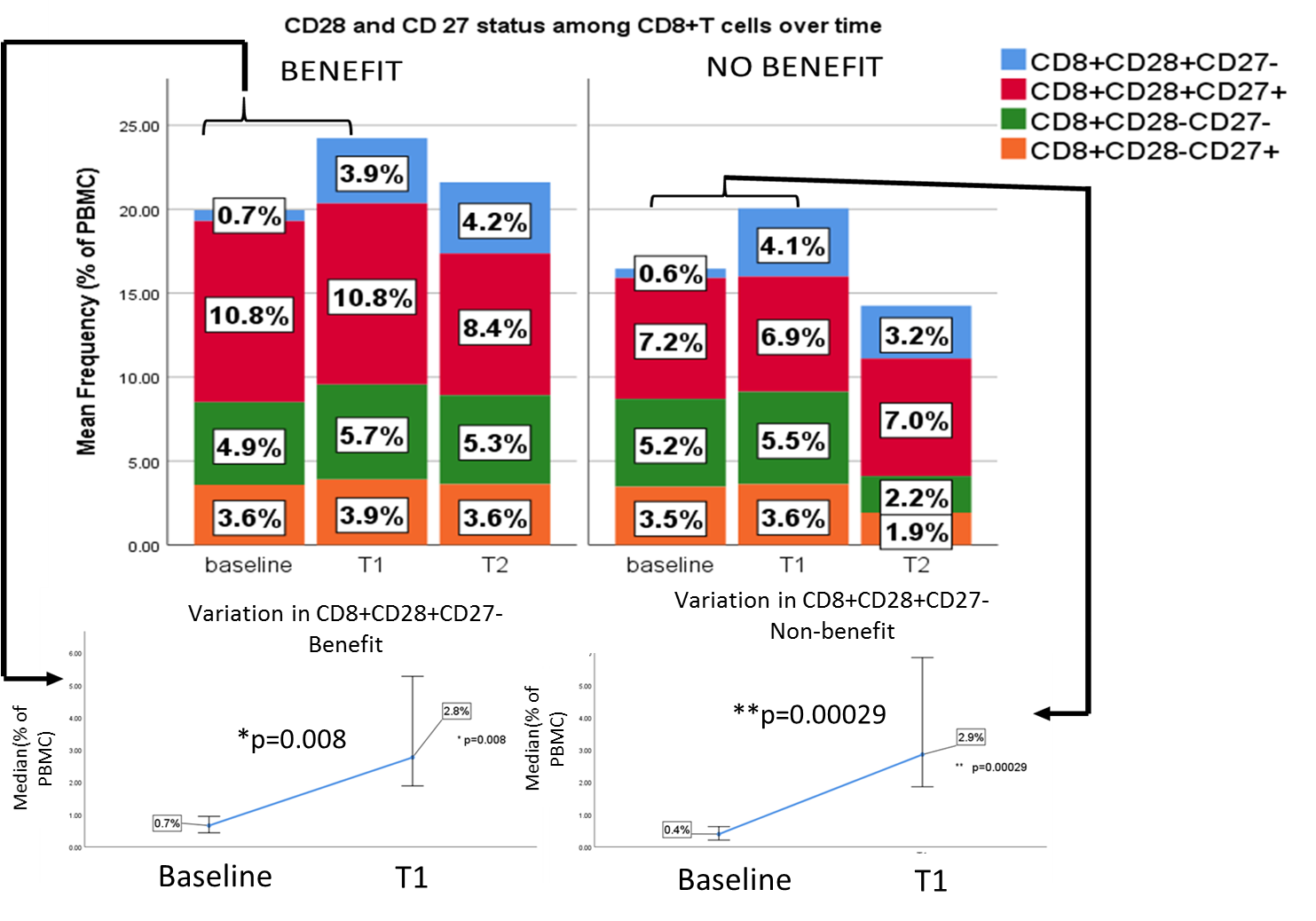


Supplementary Figure 6. Expression of CD28 among CD8^+^T cells increases over time during ICI, regardless response pattern. Clustered bar plots represent mean values for each CD8^+^subset according to expression of CD27 and CD28. Line plots underneath depict variations in median values of the CD28^+^CD27^-^ subset.


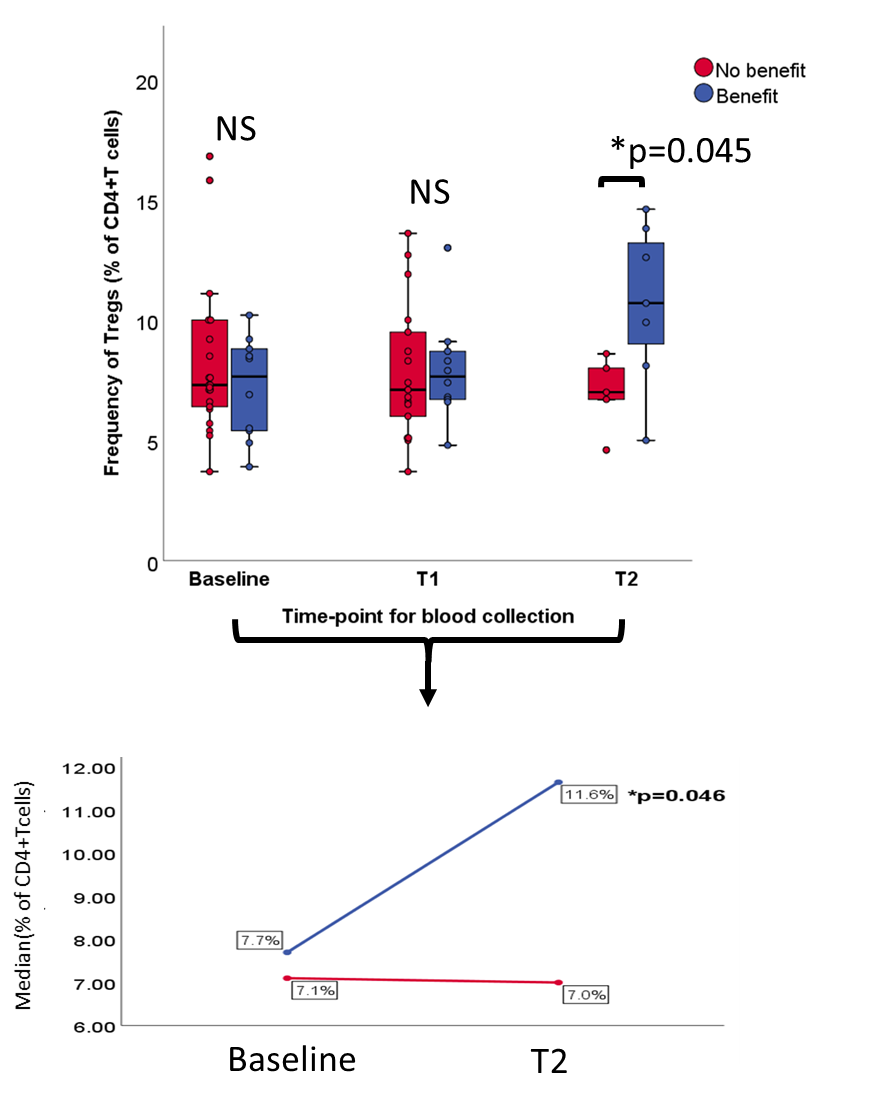


Supplementary Figure 7. Frequency of T regs (% of CD4^+^ T cells) in late follow-up samples associated with response. Line plot underneath depicts the statistically significant increase in median values of T regs among responders.

$$\boldsymbol{Immune Periphery Index=}\frac{\sqrt[\boldsymbol{3}]{\boldsymbol{CD}\boldsymbol{8}\boldsymbol{+}\boldsymbol{EM \times CD}\boldsymbol{8}\boldsymbol{+}\boldsymbol{PD}\boldsymbol{1}\boldsymbol{+}\boldsymbol{\times DC}}}{\sqrt[\boldsymbol{2}]{\boldsymbol{mMDSC \times Classical Monocytes}}}$$

Supplementary Equation 1. Mathematical expression of the Immune Periphery Index. For each patient the index is calculated by obtaining the geometric mean for relative counts (i.e. percentage of live PBMCs) of CD8+effector memory T cells (CD8+EM), CD8^+^PD1^+^ T cells and DCs as numerator and mMDSCs and classical monocytes as denominator.
